# A pilot study of an individualized comprehensive treatment for advanced gastric cancer with para-aortic lymph node metastasis

**DOI:** 10.1186/s12876-016-0422-7

**Published:** 2016-01-18

**Authors:** Qi He, Long Ma, Yang Li, Guoli Li

**Affiliations:** Research Institute of General Surgery, Jinling Hospital, School of Medicine, Nanjing University, No.305 East Zhongshan Road, Nanjing, 210002 China

**Keywords:** Advanced gastric cancer, Neoadjuvant chemotherapy, Intravenous and intra-arterial administration, D2 dissection, Radiotherapy

## Abstract

**Background:**

The incidence of the para-aortic lymph node metastasis (PALM) in patients with advanced gastric cancer is 6 to 33 %. The prognosis is poor and the 5-year survival rate is only 12 to 23 % after gastrectomy with super-extended lymph node dissection. We applied an individualized comprehensive treatment for affected patients including neoadjuvant chemotherapy via intra-arterial and intravenous administration, surgery and radiotherapy, to investigate the safety and prognostic value.

**Methods:**

Between January 2005 and December 2010, 47 advanced gastric cancer patients with PALM received 5-Fu (370 mg/m^2^) and leucovorin (200 mg/m^2^) intravenously on days 1–5, and intra-arterial infusion of etoposide (80 mg/m^2^) and oxaliplatin (80 mg/m^2^) on days 6 and 20, repeated 2 cycles. Patients achieved PR or CR of the para-aortic lymph node (PAL) were performed D2 dissection, followed by 6 cycles chemotherapy with XELOX regimen, oxaliplatin (130 mg/m^2^) on day 1 and xeloda (1000 mg/m^2^) on days 1 to 14 of a 28-day cycle, and radiotherapy to the region of PALM.

**Results:**

Forty-six patients completed 2 cycles of neoadjuvant chemotherapy. The overall response rate of the primary tumor was 80.4 % (37/46). The response rate of PAL was 76.1 % (35/46). Thirty-two patients underwent D2 dissection and six cases achieved pathological complete response (pCR). The toxicity profile was well tolerable and there was no treatment-related death. The median survival time for all patients was 23 months, and for nonsurgical and surgical patients were 12 and 29 months (*p* < 0.001), respectively. The 1-year, 2-year and 3-year survival rate was 70.96, 43.27 and 35.48 % for all patients, and for surgical patients was 96.875, 68.75, and 40.63 %.

**Conclusion:**

Advanced gastric cancer patients with PALM can obtain a survival benefit from neoadjuvant chemotherapy, subsequent surgery and radiotherapy.

**Trial registration:**

Current Controlled Trials ChiCTR-TRC-12002046.

## Background

Gastric cancer is one of the most common malignancies worldwide, and its incidence in China alone accounts for nearly half of all cases around the world [1, 2]. Most cases of gastric cancer in China are diagnosed as advanced gastric cancer [3]. Moreover, several studies indicated that more than 20 % of the patients with advanced gastric cancer show lymph node group No. 16 (para-aortic lymph node, PAL) metastasis, which is considered as distant metastasis by the UICC (Union Internationale Contre le Cancer) TNM classification and usually indicates a poor prognosis [4–7]. For these cases, gastrectomy with more radical extended lymphadenectomy, D2 plus para-aortic node dissection (PAND), has been practiced. However, several randomized clinical trials concluded that there was no survival benefit of PAND over standard D2 lymphadenectomy [8–10]. In order to improve locoregional control of gastric cancer and survival, multimodal treatment involving chemotherapy or radiotherapy in addition to surgery should be considered as a promising treatment strategy.

Survival benefits from adjuvant chemotherapy or radiotherapy have been demonstrated in previous studies [11–13]. However, the response rate of preoperative chemotherapy for advanced gastric cancer is less than 50 %. A pilot study in Japan applied a method of oral administration of S1 and local radiotherapy to treat gastric cancer with severe local infiltration and metastasis. Twelve eligible patients were enrolled. R0 resections were performed in 11 patients (91.7 %). A pathologic response was observed in ten patients (83.3 %) [14]. Based on the same theory, since 2005, we started a pilot study performing an individualized comprehensive treatment for gastric cancer patients with para-aortic lymph node metastasis (PALM), which including combination of intravenous and intra-arterial neoadjuvant chemotherapy, surgery and radiotherapy. Here we report the results of our study.

## Methods

### Ethics statement

The protocol was approved by Chinese Ethics Committee of Registering Clinical Trials, ChiECRCT and Independent Institutional Review Board (IRB) of Jinling Hospital. The trial is registered on clinicalTrialecrf.org and has the identification number ChiCTR-TRC-12002046.

### Patient eligibility

From January 2005 to December 2010, 47 out of a total of 876 hospitalized gastric cancer patients diagnosed with PALM enrolled in this study. All cases were diagnosed by endoscopic biopsy. The primary tumor, local infiltration and lymph node metastasis were valued by contrast enhanced CT scan and endoscopic ultrasound. Laparoscopy combined with peritoneal cytology was performed in patients with potentially liver and peritoneal metastases.

Eligibility criteria included: 1. Histologically proven gastric cancer; 2. Presence of para-aortic lymph node metastasis, evaluated by CT scan; 3. 35–70 years of age; 4. Performance status (PS) ≤1 on the Eastern Cooperative Oncology Group (ECOG) scale [15]; 5. no prior anti-tumor therapy; 6. Agreed to accept intravenous and intra-arterial chemotherapy and signed informed consent; 7. Adequate organ and bone marrow functions.

Exclusion criteria included: 1. Distant hematogenous metastasis; 2. Peritoneal dissemination; 3. Acute perforation, massive hemorrhage and complete obstruction which needed emergency services; 4. Serious or uncontrolled systemic diseases; 5. Chemotherapy drug allergies; 6. Pregnant or lactating; 7. With other malignancies.

The 6th edition UICC TNM classification is used for preoperative staging, and the number of lymph node stations was determined according to the Japanese Gastric Cancer Association (JGCA) classification [16].

### Treatment protocol

All patients received 2 cycles of neoadjuvant chemotherapy with a regimen of 5-Fu/leucovorin/etoposide/oxaliplatin combinations via intravenous and intra-arterial administration. 5-Fu (370 mg/m^2^) and leucovorin (200 mg/m^2^) was administered by intravenous infusion on day 1–5. Intra-arterial administration of etoposide (80 mg/m^2^) and oxaliplatin (80 mg/m^2^) was performed by Seldinger method on day 6 and 20, the catheter was inserted through femoral artery into the celiac artery and the chemicals were injected initially at relatively high doses, followed by 14 days’ rest.

**Fig. 1:**
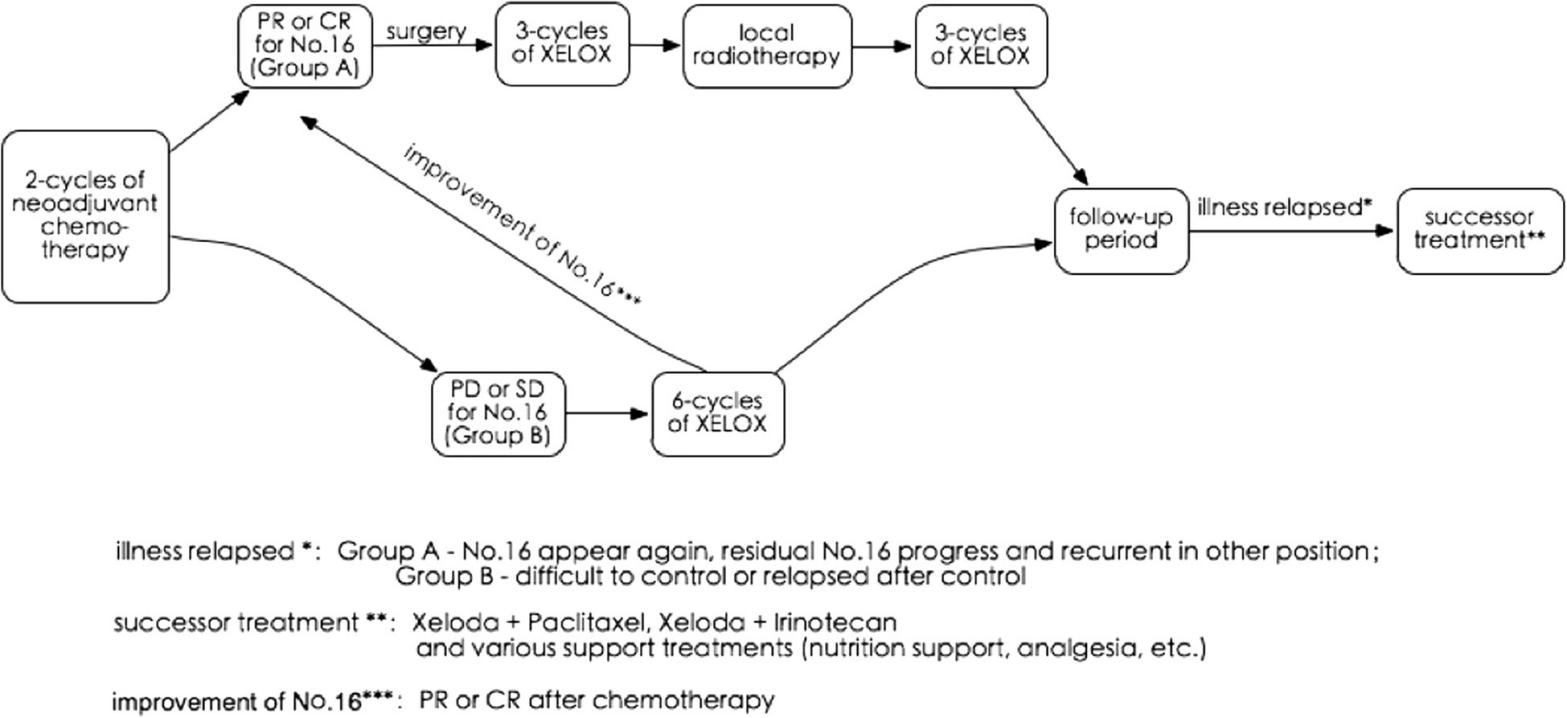
Treatment programs process

After 2 cycles of neoadjuvant chemotherapy, the chemotherapeutic response was evaluated using contrast-enhanced CT scan by two experienced radiologists, who were blinded to any of the clinical data independently, according to the Response Evaluation Criteria in Solid Tumors (RECIST) guidelines [17]. Patients were then divided into group A with complete response (CR) or partial response (PR) of PAL, or group B with stable disease (SD) or progressive disease (PD). Patients of group A underwent gastrectomy with D2 lymph node dissection, followed by 6 cycles chemotherapy with the regimen of XELOX, oxaliplatin (130 mg/m^2^) on day 1 and xeloda (1000 mg/m^2^) on days 1 to 14 of a 28-day cycle, and radiotherapy to the region of PALM. Patients of group B continued chemotherapy and radiotherapy when necessary without surgery. Treatment programs seen in Fig. 1.

For radiotherapy, blood routine test, hepatic and renal function test and CT scan were performed before radiotherapy. Gross tumor volume (GTV) was determined based on CT tumor visualizations before the first treatment. Planning target volumes (PTV) were GTV plus a 2–3 mm margin. Total doses ranged from 45 to 51 Gy (median 48 Gy).

### Evaluation and statistic analysis

Tumor responses to preoperative chemotherapy were evaluated using RECIST, based on CT scanning [17]. The histological response was evaluated according to the histological criteria of the JCGC [18]. Briefly, histological evaluation was based on the proportion of the tumor affected by degeneration or necrosis: G3, no viable tumor cells remain; G2, viable tumor cells remain in less than 1/3 of the tumor area; G1, viable tumor cells remain in more than 1/3 but less than 2/3 of the tumor area; G0, no evidence of treatment effect. All the adverse events during chemotherapy and postoperative morbidity were recorded. National Cancer Institute Common Toxicity Criteria version 3.0 was applied to assess chemotherapy-related toxicity.

All patients were followed up according to the institutional protocol. Tumor markers including serum carcinoembryonic antigen (CEA), CA19-9 were examined every 3 months. Chest X-ray and abdominal/pelvic CT scan were performed every 6 months. Gastroscopy was also required each year. PET scan was suggested when recurrence was suspected. The frequency of follow-up is trimonthly in the first 2 years and every six months after two years. Overall survival (OS) was defined as the interval from the initial date of neoadjuvant chemotherapy to the date of death from any cause. The 1 year, 2 year and 3 year survival rate was calculated using the Kaplan-Meier method. Statistical analysis was performed with SPSS version 19.0 (SPSS Inc., USA).

## Results

Between January 2005 and December 2010, 47 patients, out of a total of 876 hospitalized gastric cancer patients, including 33 men and 14 women, enrolled into the present study. The characteristics of these patients are shown in Table 1. Among 47 patients, nine patients had Borrmann type 2 tumors, 30 patients had Borrmann type 3 tumors and eight patients had Borrmann type 4 tumors. Endoscopic ultrasonography and CT scan showed that tumor has invaded the serosa in all cases and adjacent tissues in 13 cases. All patients had regional lymph node metastasis, including four cases with N1 lymph node status, 23 cases with N2 and to 20 cases with N3.

**Table 1:** The characteristics of gastric cancer patients with PALM

Characteristic	Number of patients
Sex	
Men	33
Women	14
Surgery	
Gastrectomy	18
Distal gastrectomy	14
Macroscopic type JCGC	
Type 2	9
Type 3	30
Type 4	8
Histological type	
Adenocarcinoma	37
Mucinous cancer	4
Signet ring cancer	6
Differentiation	
High-medium	2
Low	45

*JCGC* Japanese classification of gastric carcinoma

### Response and toxicity of neoadjuvant chemotherapy

Forty-six patients completed two cycles of neoadjuvant chemotherapy, and five of them received enteral nutrition though nose intestine. Responses of chemotherapy and all adverse events are shown in Table 2. The clinical overall response rate of primary tumor (CR + PR) was 80.4 % (37/46). Seven cases (15.22 %) were SD and two patients (4.35 %) were PD. Meanwhile, the response rate of PAL according to RECIST was 76.1 % (35/46), in which eight cases were CR and 27 cases were PR.

**Table 2:** The response and effects of neoadjuvant chemotherapy

Characteristic	Number of patients	Percent (%)
Response of Primary tumor		
CR	3	6.5
PR	34	73.9
SD	7	15.2
PD	2	4.4
Response of PAL		
CR	8	17.4
PR	27	58.7
SD	9	19.6
PD	2	4.4
Adverse Events (grade 3/4)		
Anemia	3	6.5
Leukopenia	3	6.5
Thrombocytopenia	2	4.3
Hepatic inadequacy	0	0
Renal dysfunction	0	0
Nausea	6	13.0
Vomiting	5	10.9
Diarrhea	3	6.5

*CR* complete response; *PR* partial response; *SD* stable disease; *PD* progressive disease

There were no treatment-related deaths. The most frequent toxicities were nausea, vomiting and diarrhea. Among the hematological adverse events, 6.52 % of patients experienced grade 3–4 anemia and leukopenia. No abnormal results for liver or renal function tests were observed in grade 3–4.

### Surgery

Thirty-five cases were CR or PR of PAL. Thirty-two patients divided to group A and underwent D2 dissection: total gastrectomy in 16 patients, distal gastrectomy in 14 patients and total gastrectomy with splenectomy in two patients. There were three patients transferred to group B owing to unresectable primary tumor with severe infiltration to the celiac artery and hepatoduodenal ligament. All patients had recovered and been discharged.

### Pathological response

In 32 postoperative resected specimens, G1, G2, and G3 response were observed in 11 cases, 15 cases and six cases, respectively.

### Local radiotherapy

Thirty-one patients in group A received radiotherapy. The region of PAL was evaluated again by CT scan before radiotherapy. One patient’s PAL with CR after neoadjuvant chemotherapy appeared again, so 25 patients out of 31 remained PALM. After radiotherapy, six cases were CR, 15 cases were PR, four cases were SD and no PD. In group B, one patient quitted after 4 cycles of chemotherapy. Five patients underwent radiotherapy because of severe back pain. There were no treatment related deaths.

### Follow-up and survival

**Fig. 2:**
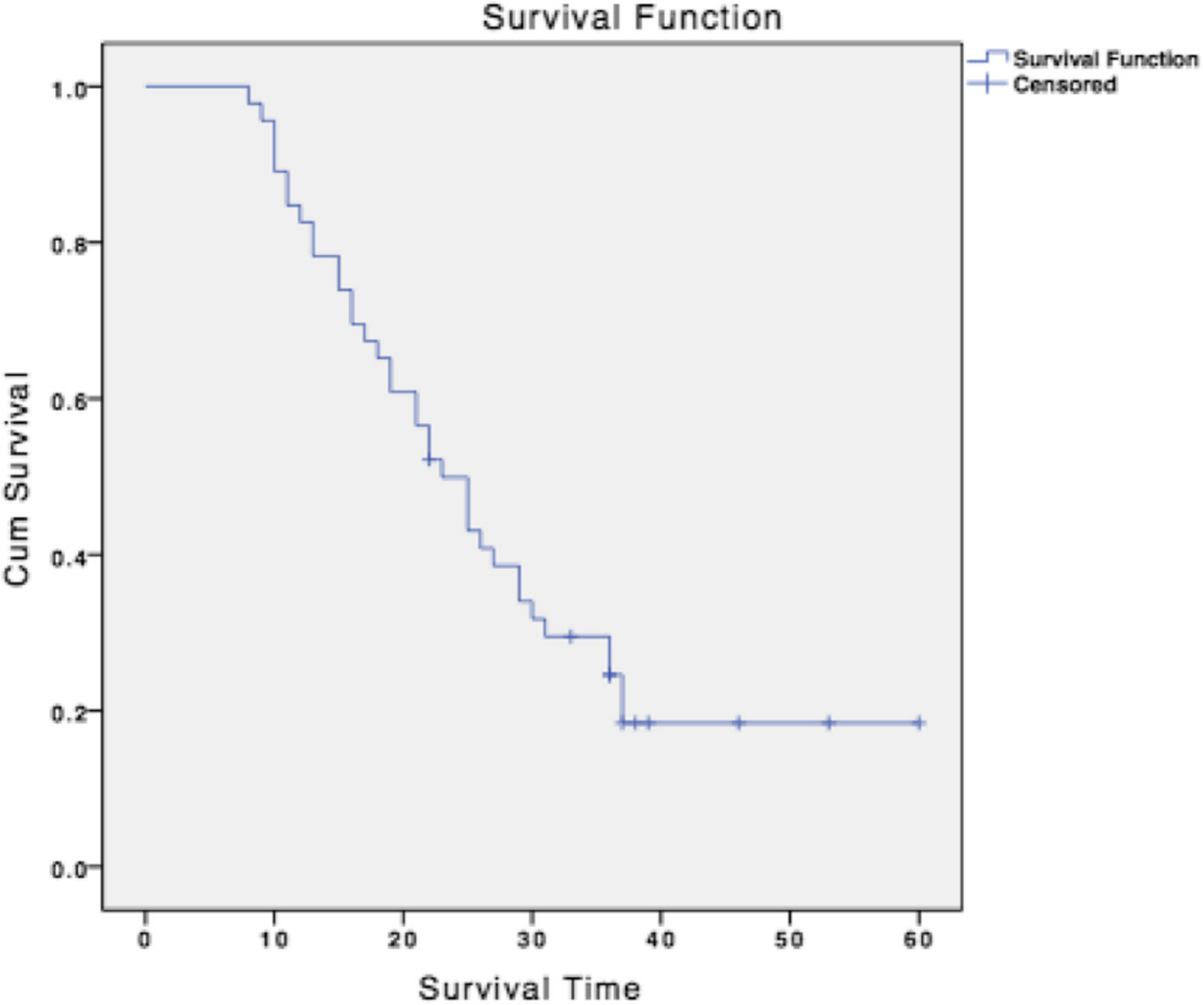
Kaplan-Meier overall survival for all patients

**Fig. 3:**
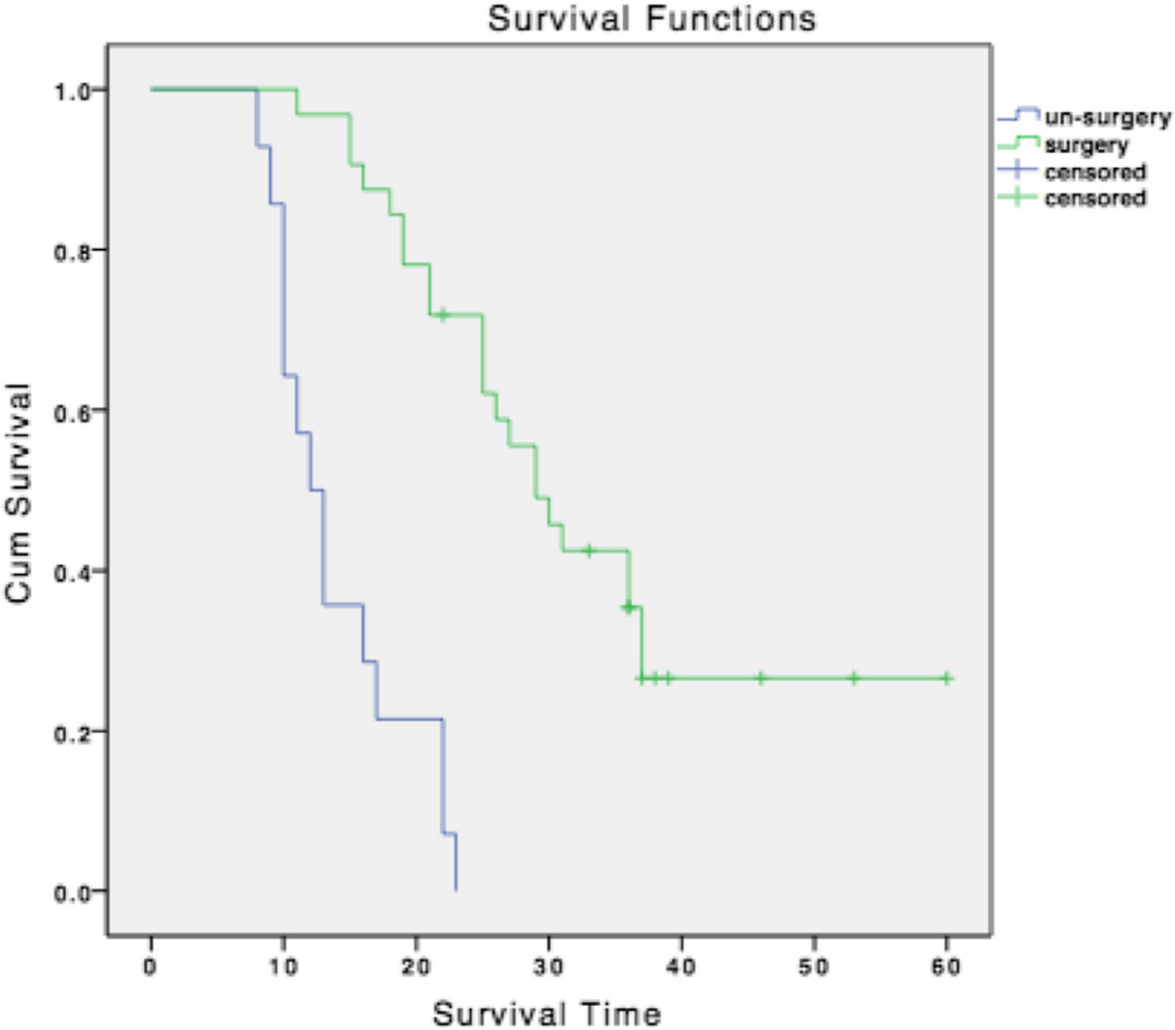
Kaplan-Meier survival curves for patients in group A and group B

By March 2013, nine patients were still alive, and the longest survival time is 59 months. The survival curves for all patients, patients in group A and group B are shown in Figs. 2 and 3. The median survival time for all patients was 23 months (95%CI, 18.686–27.314). And the median survival time for group A and group B were 29 months (95%CI, 23.624–34.376 *p* < 0.001) and 12 months (95%CI, 9.555–14.445 *p* < 0.001), respectively. The 1-year, 2-year and 3-year survival rate was 70.96, 43.27 and 35.48 % for all patients, and was 96.87, 68.75 and 40.63 % for patients in group A.

## Discussion

Para-aortic lymph nodes metastasis (PALM) is the last-tier lymph nodes for intra-abdominal metastasis of gastric cancer. The incidence of metastasis in the para-aortic lymph nodes (PLA) was 6 to 33 %. The 5-year survival for these patients is only 12 to 23 %. There is no survival benefit of aggressive surgical treatment, D2 plus para-aortic lymph node dissection [8–10]. Recently, several clinical trials for the treatment of advance gastric cancer have been carried out in Japan [15, 16], but there’s none for patients with PALM. To improve the survival of patients with PALM, it is necessary to try new therapeutic strategies, including preoperative chemotherapy and irradiation. Therefore, our study here may provide a practical treatment strategy for affected patients.

In most of the phase III clinical trails, the response rate of preoperative chemotherapy for advanced gastric cancer is less than 50 %. A pilot study in Japan applied the method of oral administration of S1 and local radiotherapy to treat gastric cancer with severe local infiltration and metastasis, which showed a response rate of 83.3 % for 12 patients and the 3-year survival rate was 58.3 % [14]. Our neoadjuvant chemotherapy approach via intra-arterial injection is based on the same theory, which strengthens local effects on the primary tumor on the basis of systemic administration. The response rate was 80.4, and 68.1 % cases achieved R0 resection. The overall median survival time was 23 months. The toxicity profile of this regimen was well tolerable and all patients with CR or PR were able to safely undergo surgery.

Since the 1980s, gastrectomy with more radical extended lymphadenectomy (D3, superextended lymphadenectomy) has been practiced at many specialized centers in Japan. However, patients would be too weak after neoadjuvant chemotherapy to perform the aggressive surgery. A randomized clinical trial JCOG0001 was conducted in Japan, the patients received two or three cycles of chemotherapy with irinotecan (70 mg/m^2^ on days 1 and 15) and cisplatin (80 mg/m^2^ on day 1), and then underwent surgery. There were three treatment-related deaths out of 55 enrolled patients and the trail was terminated, which indicated that intensive preoperative chemotherapy followed by D3 surgery is very difficult to conduct in practice [19]. Therefore, in the present study, when patients achieved complete response (CR) or partial response (PR) after 2 cycles of neoadjuvant chemotherapy, gastrectomy with D2 lymph node dissection would be performed and followed by postoperative radiotherapy to the region of PALM confirmed by CT scan. Radiotherapy was conducted even if CR of PLA achieved, because CR evaluated by CT scan doesn’t mean pathological complete response, and radiotherapy was for the management of potential micrometastasis. Resection of the primary tumor would not improve the survival when PALM exacerbated. In this case, chemotherapy should be continued and radiotherapy should be considered.

There is no doubt that gastrectomy with regional lymph node dissection contributes to improve patient outcomes, actually, gastrectomy with D2 lymphadenectomy is still the gold standard of treatment for advanced gastric cancer. However, multimodal treatment involving chemotherapy and radiotherapy in addition to surgery is now thought to be a promising treatment strategy. Survival benefits from neoadjuvant chemotherapy or radiotherapy have been demonstrated in many studies. Here, to improve the survival of advanced gastric cancer patients with PALM, we used an individualized comprehensive treatment, including neoadjuvant chemotherapy via intra-arterial and intravenous administration, surgery and radiotherapy. The results of our study demonstrate the individualized comprehensive treatment was safe and effective and can benefit advanced gastric cancer patients with PALM.

## Conclusion

Gastric cancer patients with PALM can obtain a survival benefit from neoadjuvant chemotherapy, subsequent surgery and radiotherapy.

## Abbreviations

CR: complete response
PALM: para-aortic lymph node metastasis
PD: progressive disease
PR: partial response
SD: stable disease
XELOX: oxaliplatin & xeloda

## References

[1] Lin Y, Ueda J, Kikuchi S, Totsuka Y, Wei WQ, Qiao YL (2011). Comparative epidemiology of gastric cancer between Japan and China. World J Gastroenterol.

[2] He J, Gu D, Wu X, Reynolds K, Duan X, Yao C (2005). Major causes of death among men and women in China. N Engl J Med.

[3] Wu AW, Ji JF, Yang H (2010). Long-term outcome of a large series of gastric cancer patients in China. Chin J Cancer Res.

[4] Isozaki H, Okajima K, Fujii K, Nomura E, Lzumi N, Mabuchi H (1999). Effectiveness of paraaortic lymph node dissection for advanced gastric cancer. Hepato-gastroenterology.

[5] Takashima S, Kosaka T (2005). Results and controversial issues regarding a para-aortic lymph node dissection for advanced gastric cancer. Surg Today.

[6] Sobin LH, Wittekind C (2002). TNM classification of malignant tumors.

[7] Di Leo A, Marrelli D, Roviello F, Bemini M, Minicozzi A, Giacopuzzi S (2007). Lymph node involvement in gastric cancer for different tumor sites and T stage: Italian Research Group for Gastric Cancer (IRGGC)experience. J Gastrointest Surg.

[8] Kulig J, Popiela T, Kolodziejczyk P, Sierzega M, Szczepanik A (2007). Standard D2 versus extended D2 (D2+) lymphadenectomy for gastric cancer: an interim safety analysis of a multicenter, randomized, clinical trial. Am J Surg.

[9] Yonemura Y, Wu CC, Fukushima N, Honda I, Bandou E, Kawamura T (2006). Operative morbidity and mortality after D2 and D4 extended dissection for advanced gastric cancer: a prospective randomized trial conducted by Asian surgeons. Hepatogastroenterology.

[10] Yonemura Y, Wu CC, Fukushima N, Honda I, Bandou E, Kawamura T (2008). Randomized clinical trial of D2 and extended paraaortic lymphadenectomy in patients with gastric cancer. Int J Clin Oncol.

[11] Macdonald JS, Smalley SR, Benedetti J, Hundahl SA, Estes NC, Stemmermann GN (2001). Chemoradiotherapy after surgery compared with surgery alone for adenocarcinoma of the stomach or gastroesophageal junction. N Engl J Med.

[12] Cunningham D, Allum WH, Stenning SP, Thompson JN, Van de Velde CJ, Nicolson M (2006). Perioperative chemotherapy versus surgery alone for resectable gastroesophageal cancer. N Engl J Med.

[13] Sakuramoto S, Sasako M, Yamaguchi T, Kinoshita T, Fujii M, Nashimoto A (2007). Adjuvant chemotherapy for gastric cancer with S-1, an oral fl uoropyrimidine. N Engl J Med.

[14] Inoue T, Yachida S, Usuki H, Kimura T, Hagiike M, Okano K (2012). Pilot feasibility study of neoadjuvant chemoradiotherapy with S-1 in patients with locally advanced gastric cancer featuring adjacent tissue invasion or JGCA bulky N2 lymph node metastases. Ann Surg Oncol.

[15] Oken MM, Creech RH, Tormey DC, Horton J, Davis TE, McFadden ET (1982). Toxicity and response criteria of the Eastern Cooperative Oncology Group. Am J Clin Oncol.

[16] Japanese Gastric Cancer A (1998). Japanese classification of gastric carcinoma - 2nd English edition. Gastric Cancer.

[17] Iida T, Hirata N, Hirakawa M, Noguchi T (2003). Preoperative intraarterial infusion chemotherapy for advanced gastric cancer--a retrospective review of four cases. Radiat Med.

[18] Therasse P, Arbuck SG, Eisenhauer EA, Wanders J, Kaplan RS, Rubinstein L (2000). New guidelines to evaluate the response to treatment in solid tumors. European Organization for Research and Treatment of Cancer, National Cancer Institute of the United States, National Cancer Institute of Canada. J Natl Cancer Inst.

[19] Katayama H, Ito S, Sano T, Takahari D, Mizusawa J, Boku N, et al. A Phase II study of systemic chemotherapy with docetaxel, cisplatin, and S-1 (DCS) followed by surgery in gastric cancer patients with extensive lymph node metastasis: Japan Clinical Oncology Group study JCOG1002. Jpn J Clin Oncol. 2012;42(6):556-9. 10.1093/jjco/hys054 22525210

